# Online Mendelian Inheritance in Animals (OMIA): a genetic resource for vertebrate animals

**DOI:** 10.1007/s00335-024-10059-y

**Published:** 2024-08-14

**Authors:** Imke Tammen, Marius Mather, Tosso Leeb, Frank W. Nicholas

**Affiliations:** 1 Sydney School of Veterinary Science, The University of Sydney, Sydney, NSW 2006 Australia; 2 Sydney Informatics Hub, The University of Sydney, Sydney, NSW 2006 Australia; 3 Institute of Genetics, Vetsuisse Faculty, University of Bern, Bern, 3001 Switzerland

**Keywords:** Mendelian inheritance, Likely causal variants, Phene, Web database, Ontology

## Abstract

Online Mendelian Inheritance in Animals (OMIA) is a freely available curated knowledgebase that contains information and facilitates research on inherited traits and diseases in animals. For the past 29 years, OMIA has been used by animal geneticists, breeders, and veterinarians worldwide as a definitive source of information. Recent increases in curation capacity and funding for software engineering support have resulted in software upgrades and commencement of several initiatives, which include the enhancement of variant information and links to human data resources, and the introduction of ontology-based breed information and categories. We provide an overview of current information and recent enhancements to OMIA and discuss how we are expanding the integration of OMIA into other resources and databases via the use of ontologies and the adaptation of tools used in human genetics.

## Introduction

OMIA (Nicholas et al. 2024) is a freely available, curated, online knowledgebase that provides users with up-to-date summary information on the known functional variants in vertebrate animals, together with background information on known inherited disorders and other inherited traits. OMIA is modelled on and reciprocally hyperlinked to Online Mendelian Inheritance in Man (OMIM, Online Mendelian Inheritance in Man, 2024), and provides further links to PubMed and Gene records at the National Center for Biotechnology Information (NCBI, Sayers et al. 2024), to the European Bioinformatics Institute (EBI)’s Ensembl (Martin et al. 2023) and recently introduced links to the Mondo disease ontology (Mondo, Vasilevsky et al. 2022) and the Vertebrate Breed Ontology (VBO, Mullen et al. 2024).

At the beginning of 2021, the central OMIA team increased from one to two curators, and bequest funding from 2021 onwards has enabled regular software engineering support. In this paper we provide an overview of OMIA data and a summary of recent software updates, major enhancements to likely causal variant tables and OMIA-OMIM hyperlinks, introduction of links to Mondo and VBO, and the launch of Pioneers of Mendelian Inheritance in Animals (PMIA). We also provide an update on current initiatives that focus on the use of ontologies to expand the interoperability of OMIA with other resources such as the Anstee Hub for Inherited Diseases in Animals (AHIDA, Tammen et al. 2021).

## History of OMIA

The origin and development of OMIA have been described by Nicholas (2021). Briefly, one of us (FN) began a compendium of mainly Mendelian phenes in animals (called Mendelian Inheritance in Animals, MIA) way back in 1980, in the pre-internet age. The ‘O’ was added when MIA became available on the internet on 26th May 1995. Details of the developments over the decades, and the people involved, are recorded in the Acknowledgements tab (https://omia.org/acknowledgements/) of the OMIA home page.

Until roughly five years ago, curation was predominately conducted single-handedly by one curator (FN), and limited funding restricted access to urgently needed software upgrades and modifications. The funding situation began to improve in 2017 when the Sydney Informatics Hub (SIH) started to provide University-funded bioinformatic/software-engineering resources for OMIA enhancement projects. From 2021 a bequest to the Sydney School of Veterinary Science has provided funding for the creation and continual support of AHIDA, a portal for reporting and surveillance of inherited diseases in animals in Australia. Because OMIA is a vital resource for AHIDA, some of the AHIDA funding has been allocated to SIH for the enhancement and continual maintenance of OMIA.

Since August 2010, the OMIA database and website have been using Django software, a high-level Python web framework. In July 2021, software packages were upgraded from Python 2 to Python 3 (van Rossum and Drake 2009) and from Django 1.9 to 3.2, and in 2024 to Django 4.2 (Django Software Foundation 2023), and automated tests were added with the aim of future-proofing OMIA. Site code was optimised to improve response times. Furthermore, search options were refined to increase the number of fields that are searched in a ‘quick’ search to improve user experience.

## Scope of OMIA

OMIA focuses on traits and diseases (‘phenes’) with confirmed or suspected Mendelian modes of inheritance. In addition, several phenes with unknown or complex modes of inheritance and phenes caused by somatic variants, genetically engineered modifications or genome editing are also included. While most OMIA entries are for the major domesticated animal species, more than 500 (mainly vertebrate) animal species have entries in OMIA. Information about humans and model organisms such as mouse, rat, zebrafish, and western clawed frog (xenopus) are not included, as they have dedicated species-specific resources.

As a means of highlighting and summarising advances in Mendelian inheritance in animals, OMIA also has a tab that lists Landmark papers (reporting major advances), Reviews (summarising current knowledge) and (genomic) Resource papers (reporting species maps and genome assemblies), all listed in chronological order, to provide an historical perspective.

OMIA is a globally used knowledgebase. Google Analytics user data for the past year identified 131,000 sessions by 54,000 users from 116 countries. In June 2024, OMIA included information on 2,533 phenes across 540 species, contained 5,133 phene-species entries and included a total of 32,767 references. Core statistics for key species are summarised in Table 1.

**Table 1:** Summary of OMIA phene information (accessed June 21 2024). For an up-to-date list with hyperlinks to individual entries see the summary table at the OMIA homepage: https://omia.org/home/

Traits (phenes)	Dog	Cattle^a^	Cat	Pig	Sheep	Horse	Chicken	Goat	All species
All traits: disease and non-disease	948	686	436	391	322	295	259	126	5133
All Mendelian traits: disease and non-disease	426	308	144	141	127	63	138	26	1936
• with at least one known likely causal variant	348	208	113	68	61	48	57	17	1196
• with references to genetically engineered or modified animals	5	15	2	98	12	0	11	6	237
Mendelian diseases	387	270	116	111	91	48	98	12	1403
• with at least one known likely causal variant	322	189	93	53	43	37	34	8	875

^a^ taurine cattle only, additional entries exist for indicine cattle

## Recent enhancements: text mining

Ever since PubMed started in 1997, OMIA has been conducting daily searches of all the new papers that have been entered into PubMed in the previous 24 h. Over the years, the simple keyword-based searches were improved, providing an effective but rather labour-intensive means of identifying new papers relevant to OMIA. During 2022, for example, the daily automated PubMed literature search resulted in 17,653 hits, of which 719 papers (on average 2 papers/day) were identified to be added to OMIA. In other words, the number of false positives was still depressingly high. Realising that the advent of text-mining tools should provide less labour-intensive strategies, several were tried when they became available in the early 2020s. By far the most successful turned out to be a machine-learning model developed using Microsoft’s PubMedBERT (Gu et al. 2021), which was adapted for OMIA in February 2023, and has been in daily use ever since. A total of 2,811 relevant papers were added to OMIA in the first 482 days of using the tool (average of 5.8 papers/day).

## Recent enhancements: standardised nomenclature and ontologies in OMIA

### Integration of phenotype category, *breed and disease ontologies*

Three major current projects relate to the introduction of ontologies (Table 2) for phenotype category, breed and disease definition into OMIA. Ontologies are controlled vocabularies that represent knowledge both by their meaning and their relationship to each other and provide unique numerical identifiers to enable advanced computational analysis. We aim to harmonize breed and disease definitions in OMIA in a computer-accessible format, thus enabling integration with other global online resources and integration with the submission portal of AHIDA (Tammen et al. 2021), which was launched in Australia in 2023.

**Table 2:** Phenotype, breed and disease ontologies used in OMIA

Ontology	ID	Description	Reference
Mammalian Phene Ontology	MP	An ontology of pre-coordinated phenotype terms, definitions and synonyms that can be used to describe mammalian phenotypes. It organizes phenotype terms into major biological system headers.	Smith and Eppig 2009
Mondo disease ontology	Mondo	A semi-automatically constructed ontology that merges multiple disease resources to yield a coherent merged ontology.	Vasilevsky et al. 2022
Vertebrate Breed Ontology	VBO	An ontology created to serve as a single computable resource for vertebrate breed names.	Mullen et al. 2024

**Fig. 1:**
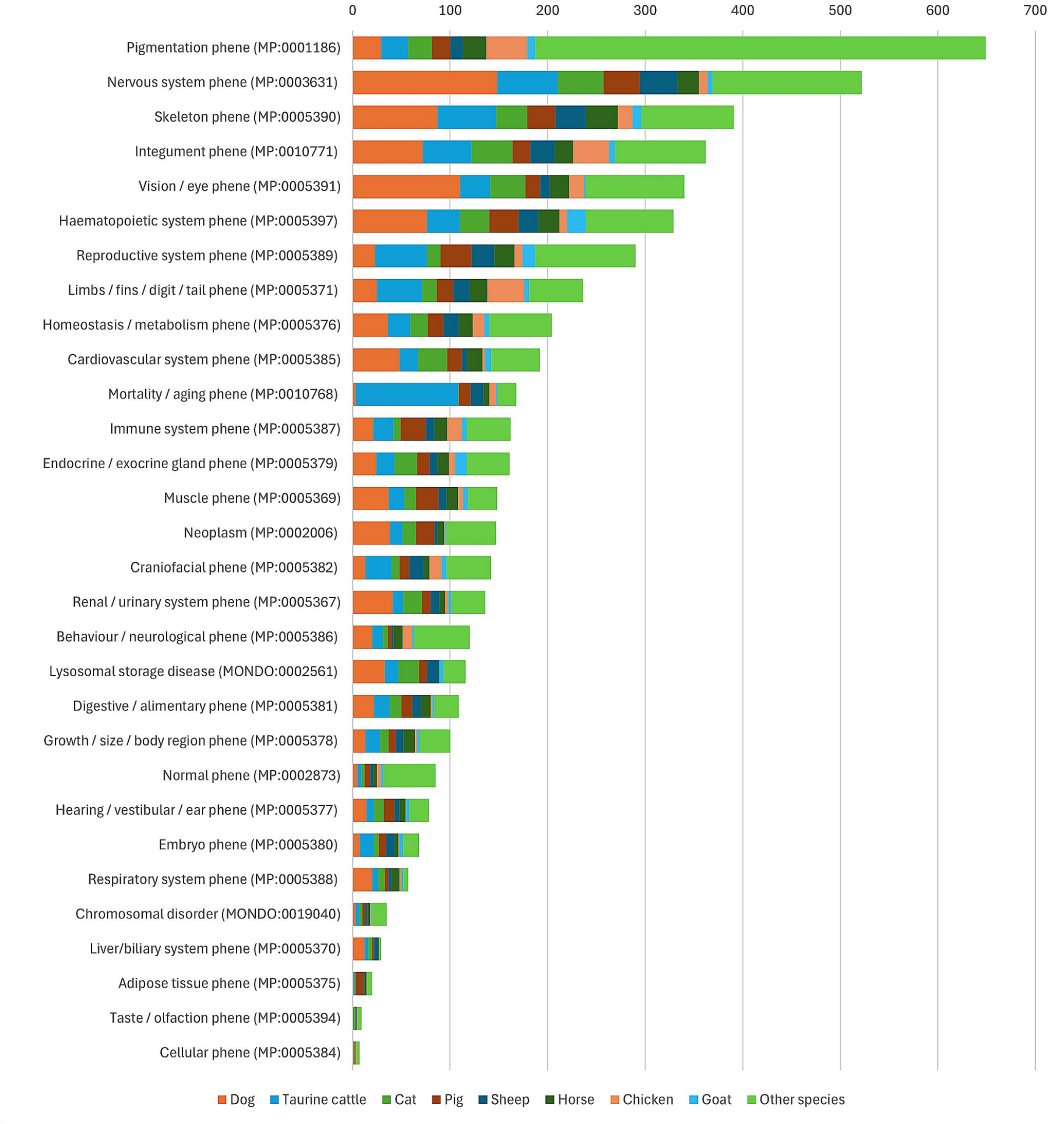
Summary of OMIA phene category information for key species (accessed 21 June 2024). Corresponding Mammalian Phenotype Ontology (MP) and Mondo identifiers are included for each OMIA category. For an up-to-date list with hyperlinks to individual entries see the OMIA ‘Browse’ page: https://omia.org/browse/

OMIA previously included a home-grown list of 20 ‘phene categories’ that could be used in ‘Advanced Search’ and in the ‘Browse’ page to create category-specific phene lists, but many OMIA phenes did not have a phene category specified. To allow for comprehensive categorisation and for easy identification of phenes based on body system, OMIA’s 20 phene categories have been replaced with 28 major biological system headers from the Mammalian Phenotype (MP) Ontology (Smith and Eppig 2009) and two headers from Mondo (Vasilevsky et al. 2022) (Fig. 1). To facilitate this, the ‘category’ field has been included in phene-species pages and each phene has been linked to at least one category by a curator (IT). Entries for the category ‘Chromosomal disorder (MONDO:0019040)’ are low as chromosomal abnormalities were previously listed in Online Cytogenetics of Animals (OCOA), hosted by the University of Sydney. This resource is no longer maintained, and we aim to transfer information to OMIA in the future.

Recognising the need to replace OMIA’s home-grown breed list with a computable comprehensive list of standardised breed names, the OMIA team instigated the creation of the Vertebrate Breed Ontology (VBO, Mullen et al. 2024) in a project led by the Monarch Initiative, in collaboration with colleagues from Iowa State University and with FAO colleagues responsible for the Domestic Animal Diversity Information System (DAD-IS). Breed information in OMIA phene-species entries and in variant tables has been replaced with links to VBO, and OMIA advanced searches allow searches by VBO breed name. Breed information is currently not entered for all phene-species or variants and is likely to be incomplete for some entries, as this update requires review of more than 5000 phene-species entries and the information is continuously changing. Currently 1297 (25%) of OMIA’s 5133 phene-species entries list at least one breed. We aim to prioritise adding this information to Mendelian phenes with known variants and variants. Of 1198 such phenes, 796 (66%) include breed information and 1194 (75%) of the 1590 likely causal variants in OMIA have at least one breed listed. It is noted that not all entries are expected to have breed information (e.g. non-domesticated species, entries relating to genetically engineered or modified animals). Recent publications reporting genotyping of large cohorts of animals for known likely causal variants (e.g. Anderson et al. 2022; Donner et al. 2023; Durward-Akhurst et al. 2024) can be useful to update breed information for known variants and this process is greatly facilitated if OMIA phene and OMIA variant and VBO breed identifiers are included.

Finally, we are working towards integrating OMIA information into Mondo (Vasilevsky et al. 2022), a global disease ontology that aims to harmonise disease definitions across the world. So far, a new field has been created in OMIA to enable addition of hyperlinks to Mondo when an OMIA phene name is a close match to a human disease term in Mondo. Currently 221 of such links have been included in OMIA. Mondo also lists terms for non-human animal diseases and to date has used OMIA as a source for 971 inherited disease terms for non-human animals. However, the non-human animal information in Mondo is still under development.

### Standardised nomenclature for likely causal variants in OMIA variant tables

The introduction of variant tables in OMIA was reported by Tammen and Nicholas (2018), who indicated that the ultimate aim was to provide an European Variation Archive (EVA) rs ID (European Variation Archive 2024) for all variants, to reduce the need to standardise and update variant information in OMIA. In 2018, OMIA listed 926 likely causal variants of which 43% had a genomic position and only 7% were listed with an EVA rs ID (Tammen and Nicholas 2018). We recognised that EVA does not accommodate all types of variants, very few authors of OMIA-relevant papers submit variant information to EVA, and new EVA IDs are allocated infrequently.

Consequently, with the help of many colleagues (see OMIA’s acknowledgement page for details: https://omia.org/acknowledgements/) we reviewed and standardised historic variant information in OMIA and applied Human Genome Variation Society (HGVS) recommendations (den Dunnen et al. 2016), with the aim to report genomic, cDNA and protein location coordinates based on a recent reference genome assembly where possible. In October 2021, variants listed in OMIA that were lacking an EVA ID but had standardised location information were submitted to EVA using a new OMIA pipeline for export of variant information in VCF for submission to EVA. Currently 436 (27%) of the 1590 likely causal variants in OMIA list an rs ID and 1241 (78%) of the variants list genomic coordinates in a reference genome. For key domesticated species (dog, cattle, cat, horse and donkey) more than 90% of the variants list coordinates in HGVS format (Table 3).

**Table 3:** Summary of OMIA variant information (accessed 21 June 2024). For an up-to-date list, see https://omia.org/results/?search_type=advanced&result_type=variant&singlelocus=yes&characterised=yes

OMIA variants	Dog	Cattle^a^	Cat	Pig	Sheep	Horse	Chicken	Goat	All species
All known likely causal variants for all Mendelian traits: disease and non-disease	528	274	195	67	88	107	72	21	1590
• All known likely causal variants for Mendelian diseases	483 (91%)	247 (90%)	147 (75%)	50 (75%)	49 (56%)	89 (83%)	39 (54%)	11 (52%)	1193 (75%)
• Variants with g. coordinates	477 (90%)	244 (89%)	184 (94%)	41 (61%)	80 (91%)	101 (94%)	45 (63%)	12 (57%)	1241 (78%)
• Variants with EVA rs ID	59 (11%)	185 (68%)	44 (23%)	30 (45%)	23 (26%)	66 (62%)	18 (25%)	8 (38%)	435 (27%)

^a^ taurine cattle only, additional entries exist for indicine cattle

Since June 2024, OMIA has been implementing the addition of NCBI-based identifiers for genomic, transcript and protein IDs to the coordinates for each variant. This process has now been completed for horse and donkey variants and is in progress for other species. However, such information is lengthy (e.g. the coordinates for the variant causing hyperkalemic periodic paralysis (HYPP) in horses are listed as NC_009154.3:g.15474228C>G; NM_001081761.1:c.4248C>G; NP_001075230.1:p.(F1416L)) and these will change when a new reference genome becomes available.

To provide unique, unchanging and easy-to-use identifiers for all likely causal variants in animals that can be used widely (e.g. by genotyping service providers to ensure greater transparency in relation to DNA testing), OMIA numerical variant identifiers (OMIAvariant ID) have been presented since 2021 in the first column of all OMIA variant tables. Pleasingly, several review papers have started to include OMIAvariant IDs in their tables (e.g. Anderson et al. 2022; Donner et al. 2023; Meadows et al. 2023). Information about likely causal variants is summarised in Table 3.

### Review of OMIA-OMIM hyperlinks

Since 1997, OMIA has been reciprocally hyperlinked to OMIM. Links to OMIM are created by OMIA curators when new phenes are entered into OMIA. In the past, this focused on adding OMIM phenotype identifiers (IDs), while OMIM gene IDs were rarely added. OMIM automatically downloads OMIA phene IDs that have an OMIM ID link once a week and updates OMIM accordingly. In OMIA, separate fields for ‘OMIM phene’ and ‘OMIM gene’ hyperlinks were recently created, and we reviewed OMIA-OMIM hyperlinks for all OMIA phenes for which a likely causal variant has been identified in at least one species. OMIM links were confirmed, deleted, or added. This review resulted in confirmation of 607 OMIM links, addition of 683 OMIM links and deletion of 46 OMIM links. Most of the added OMIM links were OMIM gene IDs (*n* = 493), as these were in the past not routinely added to OMIA. OMIA currently lists 2769 models of human traits based on links to OMIM and 2228 OMIM entries have a link to OMIA. However, a large list of OMIA phenes without known likely causal variants have not yet been reviewed, as it is more speculative to identify homology between human and animal phenes if the likely causative gene has not yet been identified. The revision of OMIA-OMIM hyperlinks and the above-mentioned links to Mondo will facilitate comparative-medicine-related research approaches.

## Recent enhancements: PMIA

In 2022, Pioneers of Mendelian Inheritance in Animals (PMIA) was added to OMIA, accessible from the home page. This project comprises a series of commentaries on papers that illustrate the early discoveries of Mendelian inheritance in animals. PMIA was first announced on Mendel Day (8 March) in 2022, and launched as part of OMIA on the 8th of July 2022, two weeks before the bicentenary of Mendel’s birth on the 22nd. Currently PMIA includes detailed commentaries by FN on 15 papers that illustrate the early discoveries of Mendelian inheritance in animals. Included are the first report of Mendelian inheritance in non-rodent animals, namely the inheritance of five trait pairs in chickens (including some drawings by Bateson) and the first reports in cattle and goats (all in 1902); followed by the first reports in guinea pigs, rabbits and fish (1903); cats (1904); and sheep (1905).

## Future enhancements under development

### Development of ontology-based clinical synopses in OMIA

Due to the large number of known single gene diseases in humans (OMIM currently lists 6,474 single gene diseases and traits) and the increasing number of known single gene diseases in non-human animals, it can be very challenging for medical clinicians and veterinarians to diagnose rare inherited diseases. In human medicine the term “diagnostic odyssey” is used to highlight that patients frequently undergo unnecessary tests and procedures and experience lengthy delays in getting a correct diagnosis and access to effective care. Tools have been developed to assist medical professionals to more efficiently identify lists of differentials for suspected inherited diseases based on clinical presentation of their patients to shorten this diagnostic odyssey. One of these tools is Phenomizer (Köhler et al. 2009, 2019), which, in essence, matches a set of clinical signs coded as human phene ontology (HPO) concepts with documented inherited phenes in compendia such as Online Mendelian Inheritance in Man (OMIM) to provide a list of ranked differentials. To enable such automated matching OMIM includes a clinical synopsis in addition to free text inforamtion for each disease. The clinical synopsis is a list of ontology-based terms that describe the disease.

To enable the development of a similar tool for veterinarians, we are aiming to include ontology-based clinical synopses to OMIA and are currently implementing the use of PhenoTagger (Luo et al. 2021) to facilitate this process. This tool uses text-mining algorithms and machine learning to extract concepts from unstructured text, such as exists in current OMIA curation fields for clinical signs and pathological findings and published papers, which are then compared with a dictionary of concepts embodied in phene ontologies such as the Human Phenotype Ontology (HPO). In other words, the unstructured text can be turned into short lists of computerised, standardised words in ontologies, depicting particular phenes. We are currently training PhenoTagger using the cross-species phenotype ontology upheno2 (Matentzoglu et al. 2019) and have created curation fields that will in the near future show clinical synopsis for OMIA entries. So, by combining PhenoTagger with Phenomizer, each trained on and/or adapted to non-human and non-model-animal information such as exists in OMIA, it should be possible to use these tools to greatly assist the diagnosis of isolated occurrences of “novel” clinical cases of inherited diseases in the veterinary context. Then, via the long-established reciprocal links between OMIM and OMIA (described above), combined with the existing links between OMIM and model-animal resources such as the Mouse Phenome Database (Bogue et al. 2023), it should be possible to greatly strengthen the potential of comparative biology to be applied in the diagnosis of inherited diseases across all vertebrates.

Some “novel” cases will be readily shown to be new cases of inherited diseases that have already been reported, and for which a DNA test may already exist. Others will have been reported in the same animal species but with no likely causal variant yet discovered, in which case the “novel” case, combined with relevant samples from relatives, can be added to the samples already available for research into the causal variant. Others will have been reported in other vertebrate species, in one or more of which a likely causal variant has also been reported, in which case a functional candidate causal gene will have been revealed, greatly enhancing the chance of discovering a likely causal variant in the species in which the “novel” case occurred.

### Evidence-based classification of the predicted pathogenicity of likely causal variants

While the OMIA list of likely causal variants is as up-to-date as possible, it is realised that there is substantial variation in the extent to which variants in that list are truly causal. In general, variants are added to the OMIA list if they are claimed to be likely causal in a peer-reviewed paper. The danger is that breeders and genotyping providers will interpret the presence of a variant in the OMIA list as a licence to use that variant as a diagnostic direct DNA test. To address this danger, a warning sign has been included at the top of all OMIA lists of likely causal variants: “Inclusion of a variant in this table does not automatically mean that it should be used for DNA testing”.

This same problem was long-ago addressed for human likely causal variants by human geneticists. Indeed, in 2015 a set of guidelines was developed by the American College of Medical Genetics and Genomics (ACMG), the Association for Molecular Pathology (AMP) and the College of American Pathologists (CAP). Developed in the context of inherited disease, these guidelines provide evidence-based criteria for classifying likely causal variants on the basis of *all* available evidence (not just the initial publication) as ‘pathogenic’, ‘likely pathogenic’, ‘uncertain significance’, ‘likely benign’, and ‘benign’ (Richards et al. 2015). In the ensuing years, these five categories have become the global standard for classifying human likely causal variants. The criteria are under continual review, now under the umbrella of the Gene Curation Coalition (GenCC; DiStefano et al. 2022). A web-based tool, the Variant Curation Interface (VCI; Preston et al. 2022) has been developed to facilitate the classification of variants.

At its 2023 conference, the International Society for Animal Genetics (ISAG) created a Standing Committee for Animal Genetic Testing Standardisation. One of its four purposes is to enable the above global standards for human variants to be adapted and applied to non-human and non-model animal species. To this end, an ISAG Variant Pathogenicity Work Group is about to commence work. Its task will be to work in conjunction with GenCC to allocate each likely causal variant in the OMIA list to one of the above five categories, which can then be clearly indicated in the OMIA variant tables. This will be a great and significant step forward.

Another important development concerning OMIA likely causal variants is a recently published paper from a human genetics team in Toronto, Canada (Haque et al. 2024). Led by Gregory Costain, this group showed how a knowledge of OMIA variants can substantially enhance the effectiveness of pathogenicity classification of human likely causal variants. Thus, human genetics can benefit from knowledge of homologous non-human non-model animal variants; and vice versa.

The obvious way forward is to exploit every opportunity to provide seamless joint interrogation of all available data from all vertebrate species.

As Haque et al. (2024) conclude. “The degree to which the exponential growth of human and veterinary genomic datasets can be integrated and harnessed to improve variant interpretation across both contexts warrants additional study.”

## Conclusion

Our vision for the future is that in addition to summarising information about inherited conditions in animals, OMIA becomes a global repository for standardised information (including pathogenicity classification) on likely causal variants for inherited diseases and other traits. We envisage that OMIA and the Anstee Hub for Inherited Diseases in Animals (AHIDA) will become joint resources for semi-automated diagnosis and for enhancement of research effectiveness in discovering new DNA diagnostics, for rare or emerging inherited conditions in animals. A key challenge is the workload of curating and enhancing OMIA which is currently done by a small team of academics with limited funding support. It is inevitable that omissions or errors occur, and we encourage anyone to send an email to omia.admin@sydney.edu.au to suggest additions or changes.

In human medicine, phene-level tools such as PhenoTagger and Phenomizer are now being combined with (or incorporated into) genomic-level tools such as Exomiser (Cipriani et al. 2020) and Seqr (Pais et al. 2022) to further enhance the chances of discovering a likely causal variant in “novel” cases.

The potential of adapting these same combinations/incorporations of phene and genomic tools so they can be applied jointly to all vertebrate species is currently being investigated.

The overall aim is to enable clinicians and geneticists across all vertebrate species to be able to utilise the same set of tools, so as to be able to exploit to the greatest extent possible the full potential of comparative biology to the benefit of all vertebrate species.

## Data Availability

No datasets were generated or analysed during the current study.
